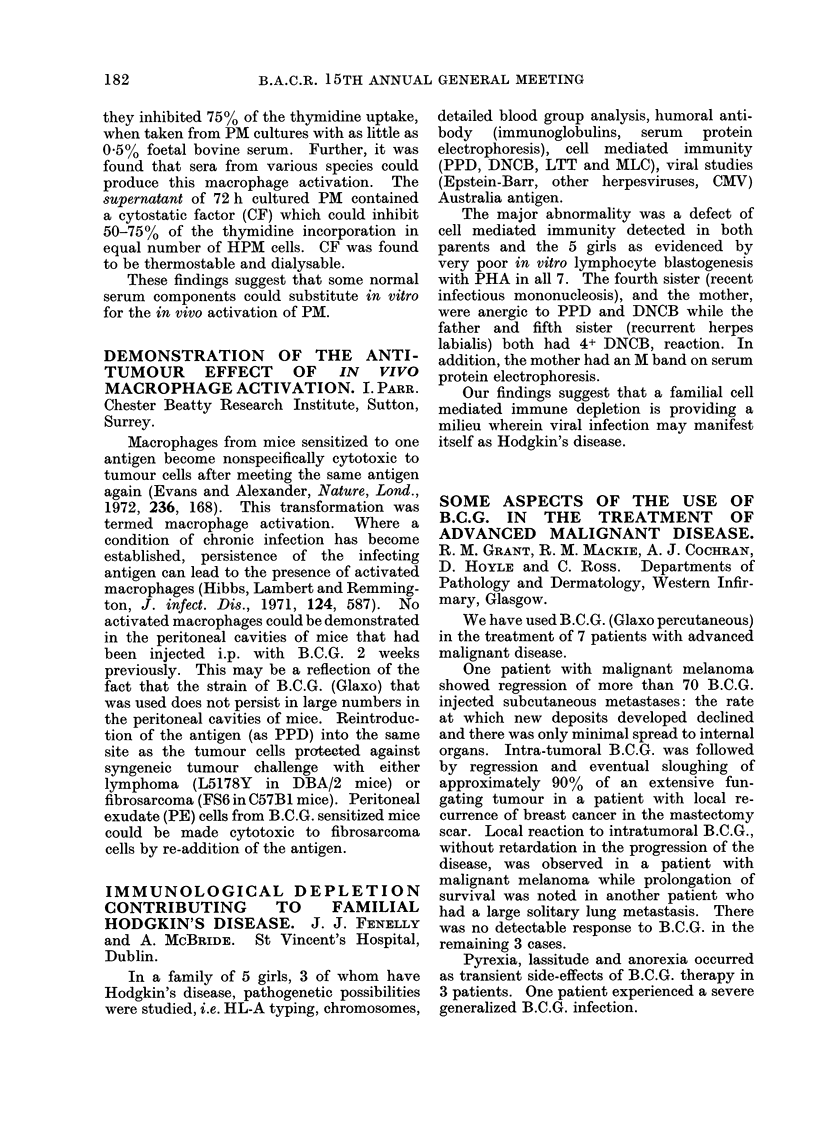# Proceedings: Demonstration of the antitumour effect of in vivo macrophage activation.

**DOI:** 10.1038/bjc.1974.162

**Published:** 1974-08

**Authors:** I. Parr


### DEMONSTRATION OF THE ANTI-TUMOUR EFFECT OF *IN VIVO* MACROPHAGE ACTIVATION.

I. PARR. Chester Beatty Research Institute, Sutton, Surrey.

Macrophages from mice sensitized to one antigen become nonspecifically cytotoxic to tumour cells after meeting the same antigen again (Evans and Alexander, *Nature, Lond.*, 1972, **236**, 168). This transformation was termed macrophage activation. Where a condition of chronic infection has become established, persistence of the infecting antigen can lead to the presence of activated macrophages (Hibbs, Lambert and Remmington, *J. infect. Dis.*, 1971, **124**, 587). No activated macrophages could be demonstrated in the peritoneal cavities of mice that had been injected i.p. with B.C.G. 2 weeks previously. This may be a reflection of the fact that the strain of B.C.G. (Glaxo) that was used does not persist in large numbers in the peritoneal cavities of mice. Reintroduction of the antigen (as PPD) into the same site as the tumour cells protected against syngeneic tumour challenge with either lymphoma (L5178Y in DBA/2 mice) or fibrosarcoma (FS6 in C57Bl mice). Peritoneal exudate (PE) cells from B.C.G. sensitized mice could be made cytotoxic to fibrosarcoma cells by re-addition of the antigen.